# NEAT expression is associated with tumor recurrence and unfavorable prognosis in colorectal cancer

**DOI:** 10.18632/oncotarget.4737

**Published:** 2015-08-07

**Authors:** Yunlong Li, Yaohui Li, Wenping Chen, Fenfei He, Zhaobang Tan, Jianyong Zheng, Weizhong Wang, Qingchuan Zhao, Jipeng Li

**Affiliations:** ^1^ Department of Gastrointestinal Surgery, State Key Laboratory of Cancer Biology, Xijing Hospital of Digestive Diseases, The Fourth Military Medical University, Xi’an, China

**Keywords:** NEAT1, colorectal cancer, relapse, prognosis

## Abstract

Long noncoding RNAs (lncRNAs) have recently been identified to be involved in various diseases including cancer. NEAT1 is a recently identified lncRNA with its function largely unknown in human malignancy. In the present study, we investigated NEAT1 expression in 239 cases of clinical colorectal cancer specimens and matched normal tissues. Statistical methods were utilized to analyze the association of NEAT1 with clinical features, disease-free and overall survival of patients. Results showed that NEAT1 expression in colorectal cancer was up-regulated in 72.0% (172/239) cases compared with corresponding normal counterparts, and related to tumor differentiation, invasion, metastasis and TNM stage. Kaplan-Meier analysis proved that NEAT1 was associated with both disease-free survival and overall survival of patients with colorectal cancer that patients with high NEAT1 expression tend to have unfavorable outcome. Moreover, cox’s proportional hazards analysis showed that high NEAT1 expression was an independent prognostic marker of poor outcome. These results provided the first evidence that the expression of NEAT1 in colorectal cancer may play an oncogenic role in colorectal cancer differentiation, invasion and metastasis. It also proved that NEAT1 may serve as an indicator of tumor recurrence and prognosis of colorectal cancer.

## INTRODUCTION

The whole-genome sequencing revealed that more than 90% of the human genome is transcribed to generate an extraordinary range of non-protein-coding (noncoding) RNAs (ncRNA) [1, 2]. These ncRNAs can be classified into two groups depending on the nucleotide size. MicroRNAs (miRNAs) are approximately 18–25 nucleotides in length. While long noncoding RNAs (lncRNAs) are longer than 200 nucleotides in length and without protein-coding function [3, 4]. Despite less well understanding of the lncRNAs compared with microRNAs, lncRNAs are found throughout the human genome [5, 6]. Originally, lncRNAs were considered as spurious transcriptional noise without biomedical functions due to the lack of protein coding capability [7]. However, lncRNAs have been validated by recent studies to be involved in various human physiological and pathophysiological processes for they may serve as powerful regulatory factors of gene function and cellular processes including gene imprinting, alternative splicing, genome rearrangement, chromatin modifications, cell growth, apoptosis and nuclear-cytoplasmic trafficking [8–12]. In addition, increasing evidence indicated that lncRNAs could participate in a wide range of signal pathways and act as either oncogene or tumor suppressor depending on their targets. Therefore, lncRNAs might have complex and extensive functions in the carcinogenesis and progression of human malignancies [13–15]. In recent reports, several lncRNAs have been identified to be abnormally expressed in various cancers and were associated with tumor cell proliferation, growth, apoptosis, invasion and metastasis [16–18].

The nuclear paraspeckle assembly transcript 1 (NEAT1) is a newly identified nuclear-restricted long noncoding RNA, which has two isoforms: 3.7 kb NEAT1_1 and 23 kb NEAT1_2. It localizes exclusively to a subnuclear structure called paraspeckles and serves as an architectural component [19–21]. The paraspeckles has been considered to be involved in regulating gene expression by retaining mRNAs for editing in the nucleus. This association suggests that NEAT1 might play a significant role in regulation of gene and consequent physiological and pathophysiological processes [22, 23]. Previous study showed that reduced expression of the nuclear long noncoding RNA NEAT1 was associated with myeloid differentiation in acute promyelocytic leukemia cells [23]. In human breast cancer, induction of NEAT1 in hypoxia could lead to accelerated tumor cell proliferation and reduced apoptosis, both of which would contribute to tumorigenesis [21]. In addition, NEAT1 was also demonstrated to drive oncogenic growth and tumor progression of prostate cancer by altering the epigenetic landscape of target gene promoters to favour transcription [24]. However, till now, reports on the role of NEAT1 in human malignancies were still limited. And little is known about the expression pattern and clinical significance of NEAT1 in colorectal cancer.

In the present study, we investigated the expression level of NEAT1 in clinical colorectal specimens and normal control tissues, as well as analyzed its association with disease-free survival and overall survival of patients.

## RESULTS

### NEAT1 expression detected in clinical specimens

**Figure 1:**
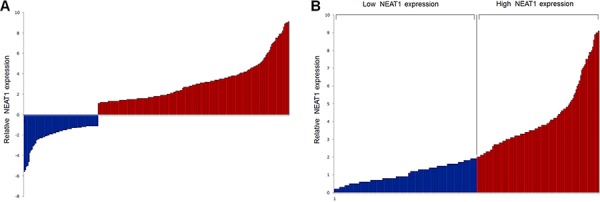
Relative expression of NEAT1 detected and subgroup classification **A.** Relative expression of NEAT1 in colorectal cancer specimens (*n* = 239) in comparison with corresponding non-tumor normal tissues (*n* = 239). NEAT1 expression was examined by real-time PCR and normalized to ACTIN expression. Results were presented as the fold-change in tumor tissues relative to normal tissues. The red column was defined as overexpression. **B.** According to the relative expression of NEAT1 in tumor tissues, tumors were classified into two groups: relative high-NEAT1 group (fold change ≥2, *n* = 110, red column) and relative low-NEAT1 group (fold change <2, *n* = 129, blue column). Results were presented as relative expression of NEAT1 in tumor tissues normlized to normal tissues.

The expression level of NEAT1 was detected in 239 matched colorectal cancer samples and adjacent, histological normal specimens by real-time PCR, and normalized to ACTIN. Considering NEAT1 lncRNA is comprised of NEAT1_1 and NEAT1_2, we utilized two primer pairs which were designed and verified as previously described to quantify NEAT1 RNA isoforms by real time PCR, one primer set recognizes both NEAT1_1 and NEAT1_2 (total NEAT1) while the other one recognizes only NEAT1_2 [23, 30]. Real time PCR results revealed that both total NEAT1 and NEAT1_2 expression was increased in colorectal cancer compared with that in normal specimens (*P* < 0.001). As NEAT1_1 is more abundant than NEAT1_2, we next evaluated the clinical significance of total NEAT1. According to data showed in Figure 1A, NEAT1 expression level in was significantly up-regulated in 72.0% (172/239) colorectal cancer specimens compared with corresponding normal counterparts. These results indicated that abnormal NEAT1 expression might be related to colorectal cancer pathogenesis. In order to facilitate analysis on the correlation of NEAT1 expression with clinicopathologic data, we classified patients into two groups: relative high NEAT1 expression group (*n* = 110, fold change ≥2) and relative low NEAT1 expression group (*n* = 129, fold change <2) (Figure 1B). The 10-gene panel test found that 39 (16.3%) tumors were MSI-H while 200 (83.7%) were MSS. Mutated APC, KRAS, BRAF and PIK3CA was found in 88(36.8%), 80 (33.5%), 42 (17.6%) and 35 (14.6%) tumors, respectively.

### Association of NEAT1 expression with clinicopathologic characteristics of patients

Based on the subgroup classification, we next analyzed the association of NEAT1 expression with clinicopathologic characteristics of colorectal cancer patients. The results were showed in Table 1. NEAT1 expression was found to be associated with tumor cell differentiation, depth of wall invasion, lymph node metastasis, distant metastases and TNM stage since high NEAT1 expression was more frequently to be detected in tumors with poor differentiation (*P* = 0.011), deep invasion (*P* = 0.040), lymph node metastasis (*P* = 0.026), distant metastases (*P* = 0.027) or advanced TNM stage (*P* = 0.026). While no statistically significant correlations were observed between NEAT1 expression and sex (*P* = 0.771), age at diagnosis (*P* = 0.736), tumor location (*P* = 0.792), tumor size (*P* = 0.117), MSI (*P* = 0.164), APC mutation (*P* = 0.227), KRAS mutation (*P* = 0.251), BRAF mutation (*P* = 0.112) or PIK3CA mutation (*P* = 0.154).

**Table 1:** Association of NEAT1 expression with clinical features

Variable	*n*	NEAT1 expression		*P*
		Low	High	
Total	239	129	110	
**Sex**				0.771*
Male	141	75	66	
Female	98	54	44	
**Age at diagnosis**				0.736*
≤60	144	79	65	
>60	95	50	45	
**Tumor site**				
Left colon	68	36	32	0.389*
Right colon	78	38	40	
Rectum	93	55	38	
**Tumor size**				0.117*
≤3.0 cm	82	50	32	
>3.0 cm	157	79	78	
**Differentiation status**				0.011*
Well	41	31	10	
Moderately	103	62	41	
Poor	95	46	49	
**Depth of invasion**				0.040*
T_1_+ T_2_	92	57	35	
T_3_+ T_4_	147	71	76	
**Lymph node metastasis**				0.026*
Absent	92	58	34	
Present	147	71	76	
**Distant metastasis**				0.027*
Absent	208	118	90	
Present	31	11	20	
**TNM stage**				0.026*
I+II	92	58	34	
III+IV	147	71	76	
**MSI**				0.285*
MSS	200	111	89	
MSI-H	39	18	21	
**APC mutation**				0.227*
(−)	151	86	65	
(+)	88	43	45	
**KRAS mutation**				0.251*
(−)	159	90	69	
(+)	80	39	41	
**BRAF mutation**				0.112*
(−)	197	111	86	
(+)	42	18	24	
**PIK3CA mutation**				0.154*
(−)	204	114	90	
(+)	35	15	20	

* *P* value when expression levels were compared using Pearson χ^2^ test

### Association of NEAT1 expression with disease-free survival of patients

**Figure 2:**
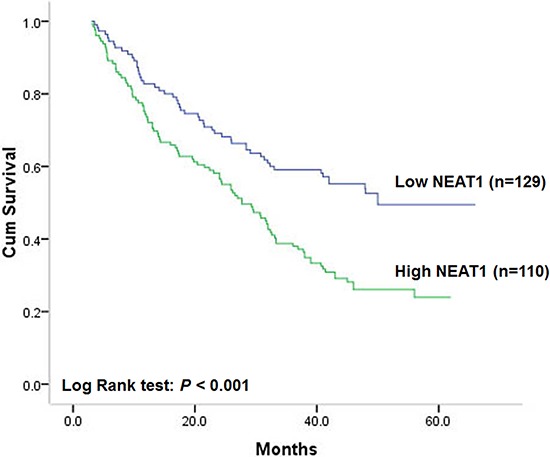
Correlation of NEAT1 expression with disease-free survival

Kaplan-Meier analysis was used to evaluate the disease-free survival of patients with colorectal cancer and NEAT1 expression. Results showed that patients with high NEAT1 expression in colorectal cancer tissues had unfavorable disease-free survival in comparison to those with low NEAT1expression (Figure 2, log-rank test: *P* < 0.001). The postoperative median disease-free survival time of patients with tumor of low NEAT1 expression was 50 months (95% CI cannot be estimated) while that of patients with tumor of high NEAT1 expression was 28 months (95% CI: 22–34). This survival pattern indicated that patients with colorectal cancer of high NEAT1 expression had a higher risk of tumor relapse compared with colorectal cancer of low NEAT1 expression. In addition, differentiation status (log-rank test: *P* = 0.012), depth of invasion (log-rank test: *P* = 0.005), lymph node metastasis (log-rank test: *P* = 0.002) and TNM stage (log-rank test: *P* < 0.001) were also proved to be associated with disease-free survival of these patients, which indicated that patients with colorectal cancer of poor differentiation, deep invasion, lymph node metastasis or advanced TNM stage had shorter disease-free survival and higher risk of relapse than those without. However, sex, age, tumor location or tumor size had no prognostic impact on disease-free survival of patients with colorectal cancer. Unadjusted hazard ratio (HR) was showed in Table 2.

**Table 2:** Association of NEAT1 and clinical factors with disease-free survival

	Unadjusted HR* (95% CI)	*P*	Adjusted HR^†^ (95% CI)	*P*
High NEAT1	1.93(1.38–2.71)	<0.001	1.80(1.27–2.55)	0.001
Sex	0.91(0.60–1.38)	0.907	0.93(0.59–1.47)	0.753
Age at diagnosis	1.18(0.85–1.64)	0.312	1.13(0.81–1.56)	0.473
Tumor site	1.22(0.81–1.86)	0.342	1.24(0.83–1.95)	0.356
Tumor size	1.49(0.95–2.36)	0.083	1.30(0.59–2.85)	0.520
Differentiation status	1.70(1.07–2.69)	0.026	1.64(1.06–2.54)	0.035
TNM stage	5.76(3.12–10.65)	<0.001	4.20(1.72–10.24)	0.002

* Hazard ratios in univariate models

† Hazard ratios in multivariable models

Abbreviations: HR, hazard ratio; 95% CI, 95% confidence interval.

To verify the independent prognostic role of NEAT1 expression on disease-free survival of patients with colorectal cancer, cox proportional hazards model adjusted for sex, age, differentiation status, TNM stage, MSI, KRAS, BRAF and PIK3CA mutation and was utilized to control for confounding factors. As a result, NEAT1 expression level was proved to be an independent prognostic factor after controlling for all these factors. Adjusted HR was 1.00 (as a reference) in low NEAT1 expression tumors, the adjusted HR of patients with colorectal cancer of high NEAT1 expression was 1.80 (95% CI: 1.27–2.55 *P* = 0.001, Table 2). These results indicated that patients with high NEAT1 expression would have higher risk to relapse than those with low level of NEAT1.

### Association of NEAT1 expression with overall survival of patients

**Figure 3:**
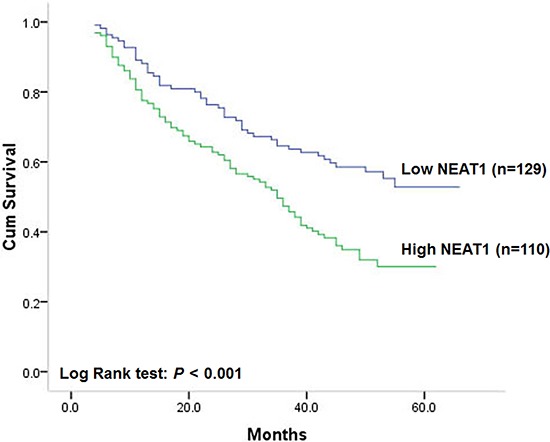
Correlation of NEAT1 expression with overall survival

Similar to the results on disease-free survival, a statistically significant association between overall survival and NEAT1 expression level was found. Kaplan-Meier analysis showed that patients with colorectal cancer of high NEAT1 expression had worse overall survival compared with patients with tumor of low NEAT1 expression (Figure 3, log-rank test: *P* < 0.001). The postoperative median overall survival time of patients with low expression of NEAT1 cannot be estimated for over 50% patients survived, while that of patients with high expression of NEAT1 was 35 months (95% CI: 29–41). Moreover, differentiation status (log-rank test: *P* = 0.005), depth of invasion (log-rank test: *P* = 0.010), lymph node metastasis (log-rank test: *P* = 0.001) and TNM stage (log-rank test: *P* < 0.001) were also proved to be prognostic factors for overall survival of patients with colorectal cancer. Patients with colorectal cancer of poor differentiation, deep invasion, lymph node metastasis or advanced TNM stage had shorter overall survival. However, sex, age, tumor location or tumor size had no prognostic value on overall survival of patients. Unadjusted HR was shown in Table 3.

**Table 3:** Association of NEAT1 and clinical factors with overall survival

	Unadjusted HR* (95% CI)	*P*	Adjusted HR^†^ (95% CI)	*P*
High NEAT1	1.88 (1.32–2.69)	<0.001	1.70 (1.18–2.45)	0.005
Sex	0.79 (0.52–1.21)	0.280	0.85 (0.54–1.36)	0.501
Age at diagnosis	1.18 (0.84–1.66)	0.347	1.17(0.82–1.67)	0.389
Tumor site	1.25 (0.71–2.22)	0.440	1.18 (0.63–2.20)	0.601
Tumor size	1.76 (0.86–3.06)	0.121	1.73 (0.82–2.96)	0.144
Differentiation status	2.24 (1.33–3.76)	0.002	2.16 (1.28–3.61)	0.005
TNM stage	6.43 (3.39–12.18)	<0.001	4.56 (1.82–11.40)	0.003

* Hazard ratios in univariate models

† Hazard ratios in multivariable models

Abbreviations: HR, hazard ratio; 95% CI, 95% confidence interval.

Multivariate analysis showed that NEAT1 expression could be a prognostic factor for overall survival of patients with colorectal cancer independent of gender, age, differentiation status, TNM stage, MSI, KRAS, BRAF and PIK3CA mutation. The adjusted HR of patients with tumors of high NEAT1 expression was 1.70 (95% CI: 1.18–2.45 *P* = 0.005, Table 3) with patients of low NEAT1 expression to be reference. In addition, lymph node metastasis and TNM stage were also showed to be independent prognostic factors after controlling for other clinicopathologic factors. However, no statistically independent correlation of overall survival with age, gender, differentiation status, depth of invasion, tumor location or tumor size and was found among patients with colorectal cancer (Table 3).

## DISCUSSION

Colorectal cancer is one of the most common malignant tumors worldwide [31, 32]. In the last two decades, the incidence of CRC in China has been increasing, ranking it the third out of all cancer-related deaths [33]. Although patients with colorectal cancer at an early stage can be cured by surgery, more than half of patients are diagnosed at advanced stage accompanied by malignant proliferation and endure high risk of tumor recurrence. Even in early stage colorectal cancer, approximately 5% to 30% of patients would develop recurrent disease and eventually die of it. Chemotherapy has been proved to could reduce tumor recurrence and benefit the life expectancy of patients with colorectal cancer. However, chemotherapy will inevitably result in significant clinical toxic and side-effect to normal organ and tissue. Thus, one of the greatest challenges in colorectal cancer management now is to accurately predict outcome so that we can determine who will benefit from adjuvant therapy. Recently, molecules involved in cancer progression have been thought to could serve as markers for prognosis. According to this concept, better understanding of the pathogenesis and identification of novel biomarkers for colorectal cancer will shed light on the understanding of the molecular mechanisms underlying cancer carcinogenesis and progression, providing more effective management strategies of colorectal cancer.

The function and clinical relevance of miRNAs was identified first and then, lncRNAs were reported more recently. Recent studies demonstrated that they could regulate gene expression at various levels, including chromatin modification, transcription and post-transcriptional processing. And lncRNAs were showed to be often expressed in a disease-, tissue- or developmental stage-specific manner pointing toward specific functions for lncRNAs in human malignancies [24, 34]. Multiple lines of evidences proved that lncRNAs dysregulation played key roles in cancer cell proliferation, survival, invasion, and metastasis by regulating gene expression, acting as either oncogene or tumor suppressor [35–37]. Therefore, identification of cancer-associated lncRNAs and clarification of their clinical impact may provide a missing piece of the well-known oncogenic and tumor suppressor network puzzle.

Therefore, we conducted the present study to determine NEAT1 expression pattern and its association with clinical features, relapse and prognosis of patients with colorectal cancer. Results proved that NEAT1expression was increased in 72.0% (172/239) colorectal cancer specimens compared with that in matched adjacent normal tissues, which indicated that the expression of NEAT1 was up-regulated in colorectal cancer compared with that in normal specimens. Statistical analysis revealed that relative NEAT1 expression in colorectal cancer was significantly associated with tumor differentiation status, depth of invasion, lymph node metastasis, distant metastasis, and advanced TNM stage for high NEAT1 expression was more frequently to be detected in colorectal cancer with poor differentiation, deep invasion, lymph node metastasis, distant metastasis, and advanced TNM stage, indicating that NEAT1 might play a oncogenic role in colorectal cancer differentiation, invasion and metastasis. However, NEAT1 expression was not found to be correlated with patients’ age, gender or tumor site. As NEAT1 was proved to be associated with colorectal cancer invasion and metastasis, which may determine tumor recurrence, we further analyzed its association with disease-free survival and overall survival of patients. In the hospital-based study cohort, high NEAT1 expression was proved to be correlated with unfavorable disease-free and overall survival. The prognostic impact of NEAT1 for disease-free and overall survival was statistically significant in not only univariate analysis but also multivariate analysis adjusted for confounding characteristics, which indicated that NEAT1 could be an independent marker of relapse and prognosis for patients with colorectal cancer. Prolongation of disease-free survival is a clinical benefit that extending disease-free survival means prevention or delay of recurrence or metastasis. In this regards, our findings suggested that measurements of NEAT1 expression may help identify patients who were in high risk of early recurrence or metastasis. So it could contribute to accurate prediction of the prognosis and recurrence probability of patients following potentially curative surgery and consequently to make tailored treatment for each individual patient, thus, prevent patients from receiving excessive or insufficient adjuvant treatment, both of which were harmful.

The functional mechanism of NEAT in cancer is still uncertain. As a critical component of the paraspeckle structure which has been demonstrated to be involved in the transcriptional regulation of gene expression, NEAT1 may have the ability to regulate its target gene directly by controlling the expression of adenosine-to-inosine hyper-edited mRNAs through the nuclear retention of target transcripts. In addition, it was also demonstrated that NEAT1 could respond to cellular cues and ligand signaling in a manner reminiscent of the coding transcriptome, indicating a role for NEAT1 beyond its interaction with paraspeckles [24]. Certainly, further studies will be needed to determine the oncogenic mechanism of NEAT1 in cancer.

Our study has several strengths. It included a hospital-based prospective study cohort with large sample size, adequate follow-up time and intimate information on clinicopathological characteristics. To avoid NEAT1 expression being affected by preoperative neoadjuvant chemotherapy, we limited the cohort to patients who had not received neoadjuvant chemotherapy. In addition, we also investigated critical molecular events such as APC, KRAS, BRAF and PIK3CA mutations and MSI, all of which have been associated with colorectal cancer prognosis to justify the prognostic role of NEAT1 [38–44].

In conclusion, we proved that NEAT1 expression in clinical colorectal cancer was associated with tumor differentiation, invasion and metastasis, indicating that NEAT1 may be a mediator for the functions of oncogene in the differentiation and progression of colorectal cancer. Our study provided the first evidence that NEAT1 was an independent prognostic factor of disease-free and overall survival for patients with colorectal cancer, suggesting that NEAT1 may be a potential predictive marker of tumor recurrence and prognosis for patients with colorectal cancer.

## MATERIALS AND METHODS

### Patients and specimens

The present research has been approved by the Ethics Committee of Fourth Military Medical University. All patients or family members involved have provided written informed consent. Briefly, fresh clinical colorectal cancer specimens as well as adjacent normal tissues were collected from 239 patients who underwent surgery between January 2008 and June 2009 in Xijing Hospital of Digestive Diseases. All the fresh tissues were obtained within 10 minutes after surgical removal and put into liquid nitrogen for 10 min, then into a −80°C ultra-freezer for mRNA isolation. The histomorphology of all tissue specimens were confirmed by the Department of Pathology, Xijing Hospital. Patients with following criteria were subsequently excluded: received treatment prior to surgery including neoadjuvant chemotherapy; harvested insufficient specimens for RNA isolation; diagnosed as colorectal stromal tumor; diagnosed with additional cancers; refused consent. Clinicopathologic information and follow-up data of the remaining 239 patients were prospectively entered into a database, which was under a close follow-up scheme and updated with respect to survival status every three month by telephone visit and questionnaire letters.

### Measurement of endpoints

Disease-free survival is defined as the time elapsed from surgery to the first occurrence of any of the following events: recurrence of colorectal cancer; colorectal cancer distant metastasis; development of second non-colorectal malignancy excluding basal cell carcinomas of the skin and carcinoma in situ of the cervix; or death from any cause without documentation of a cancer-related event. The diagnosis of recurrence and distant metastasis was based on the imaging method such as endoscope, ultrasonography, computed tomography, magnetic resonance imaging and position emission tomography, if possible, cytologic analysis or biopsy. Overall survival is defined as the time elapsed from surgery to death of patients with colorectal cancer. Death of participants was ascertained by reporting from the family and verified by review of public records. The disease-free and overall survival status was assigned by trained staff blinded to other clinicopathologic and NEAT1 expression data.

### Real-time polymerase chain reaction

Total RNA from all the 239 colorectal cancer and matched adjacent normal specimens was extracted using Trizol reagent (Invitrogen, Carlsbad, CA) according to the recommendation of manufacturer. The cDNA synthesis was performed using approximately 5 μg RNA per 20 μL using a cDNA reverse transcription kit (Fermentas). Real-time PCR was performed on an ABI 7500 system (Applied Biosystems) using SYBR Green I (TAKARA). NEAT1 primers used were: NEAT1 forward, 5′-CTTCCTCCCTTTAACTTATCCATTCAC-3′; NEAT1 reverse, 5′-CTCTTCCTCCACCATTACCAACAATAC-3′, which recognizes both NEAT1_1 and NEAT1_2 (total NEAT1); NEAT1_2 forward, 5′-CAGTTAGT TTATCAGTTCTCCCATCCA-3′; NEAT1_2 reverse, 5′-GTTGTTGTCGTCACCTTTCAACTCT-3′, which recognizes only NEAT1_2. The internal control ACTIN primers used were: Forward 5′-ATCATGTT TGAGACCTTCAACA-3′ and Reverse 5′-CATCTC TTGCTCGAAGTCCA-3′. After first strand synthesis, an equivalent of 50 ng of starting total cellular RNA (1/10 of the cDNA reaction) was added to two duplicate PCR reactions containing 12.5 μL SybrGreen mix, 0.5 μL SybrGreen rox, 100 nmol/L forward primer and 100 nmol/L reverse primer in a final volume of 25 μL. Each sample was used in a single reaction that cycled at 95°C for 10 min (to activate enzyme), followed by 45 cycles of 95°C for 10 s and 60°C for 34 s on an ABI SDS 7500 system (Applied Biosystems). The mRNA expression of NEAT1 was analyzed using the 2^−ΔΔCt^ method. Fluorescent data were converted into RQ measurements, which stand for relative expression automatically by the SDS system software and exported to Microsoft Excel. NEAT1 expression levels were normalized to ACTIN. Thermal dissociation plots were examined for biphasic melting curves, indicative of whether primer-dimers or other nonspecific products could be contributing to the amplification signal.

### DNA extraction, microsatellite instability (MSI), genetic mutation analysis

DNA was extracted from paraffin embedded tissue, MSI status was determined via testing on a 10-gene panel in tumor DNA using 10 microsatellite markers (BAT25, BAT26, BAT40, MYCL, D5S346, D17S250, ACTC, D18S55, D10S197, and BAT34C4) as described in previous study [25]. In brief, tumors with MSI-high/ microsatellite stability (MSI-H) was defined if instability was observed for ≥30% of markers, while and MSI-low/microsatellite stability (MSS) was defined if instability was observed for <30% of the markers. And we also performed PCR and pyrosequencing targeted for APC (codons 1286–1520 of exon 15), KRAS (codons 12 and 13), BRAF (codon 600) and PIK3CA (exons 9 and 20) [26–29].

### Statistical analysis

Statistical analysis was carried out by the statistical package SPSS (version l3.0). Associations between NEAT1 expression and categorical variables were analyzed by Pearson χ^2^ test. Correlation coefficients were analyzed by contingency or Spearman correlation analysis, as appropriate. Survival curves were estimated using the Kaplan-Meier method, and differences in survival distributions were evaluated by the log-rank test. Cox’s proportional hazards modeling of factors potentially related to survival was performed in order to identify which factors might have a significantly independent influence on survival. Differences with a *P* value of 0.05 or less were considered to be statistically significant.
